# Commentary: Maternal immune activation evoked by polyinosinic: polycytidylic acid does not evoke microglial cell activation in the embryo

**DOI:** 10.3389/fncel.2016.00041

**Published:** 2016-02-23

**Authors:** Hans-Gert Bernstein, Yael Piontkewitz, Gerburg Keilhoff

**Affiliations:** ^1^Department of Psychiatry, University of MagdeburgMagdeburg, Germany; ^2^George S. Wise Faculty of Life Sciences, Strauss Center for Computational Neuroimaging, Tel Aviv UniversityTel Aviv, Israel; ^3^Department of Biochemistry and Cell Biology, University of MagdeburgMagdeburg, Germany

**Keywords:** maternal immune activation, Poly I:C, Microglia, astrocytes, offspring

Immune-related abnormalities, which probably result from maternal infections during pregnancy, can be found in patients with schizophrenia and other mental disorders. In the endeavor to simulate this environmental schizophrenia risk in animal models, maternal immune activation (MIA) by infectious agents has been introduced (Zuckerman et al., 2003; Meyer et al., 2005). In a majority of publications on MIA, either lipopolysaccharide (LPS), a cell wall component of Gram-negative bacteria, or the viral mimetic polyriboinosinic-polyribocytidilic acid (poly I:C), are administered to pregnant mouse or rat dams. These maternal immune challenges are considered as suitable schizophrenia paradigms, since they induce characteristic (and often similar) anatomical, cellular, neurochemical, and behavioral alterations in the offspring, which are of relevance for schizophrenia (Meyer et al., 2009 and many others). MIA with either LPS or poly I:C generates a broad immune-inflammatory response in the developing CNS of the offspring (for recent reviews see Dean et al., 2015; Giovanoli et al., 2015a; Smolders et al., 2015). However, the action of both agents might differ with regard to one remarkable aspect: while LPS activates microglia *in vivo* and *in vitro* (Roumier et al., 2008; Cunningham, 2013; Dean et al., 2015; Zager et al., 2015 and others), poly I:C possibly does not (no activation: Olson and Miller, 2004; Piontkewitz et al., 2012; Giovanoli et al., 2015a,b; Smolders et al., 2015; activation: Patro et al., 2010; Juckel et al., 2011; Missault et al., 2014; Van den Eynde et al., 2014; Zhu et al., 2014). In an elegant set of *in vivo* and *in vitro* experiments Smolders et al. (2015) examined the effect of LPS and poly I:C under identical conditions. In particular, they investigated whether embryonic microglia can be directly activated by incubating mouse brain slices from embryonic day 15.5 with either saline, poly I:C, IL-6, or LPS. They found that LPS, contrary to poly I:C or IL-6, activates microglia to “a detrimental activation state.” When discussing possible pathophysiologic consequences of poly I:C's failure to activate microglia, Smolders et al. (2015) come to three conclusions: (i) It is unlikely that embryonic microglia dysfunction is the main mechanism that induces developmental abnormalities, (ii) poly I:C might evoke developmental deficits by directly acting on neuronal development, and (iii) it cannot be excluded that poly I:C effectuates an embryonic microglia priming, which results in an exaggerated response of microglia. Apart from “priming of microglia by poly I:C” being an exciting idea, there is yet little experimental evidence in favor of the existence of such a mechanism (perhaps via changes in the microglial kynurenine pathway? Giovanoli et al., 2015b). Hence, we would like to concentrate on the first two assumptions. Let's begin with the second one: is it conceivable (and plausible) that poly I:C induces developmental deficits by directly acting on neuronal development? In our opinion the answer is yes. Poly I:C is a strong agonist of Toll-like receptor 3 (TLR3). This receptor is already expressed in very immature neurons (Shi et al., 2013), and becomes up-regulated in a subpopulation of neurons after the injection of poly I:C (Deleidi et al., 2010). Moreover, poly I:C was found to depress embryonic neuronal stem cell division and population of the superficial layers of the neocortex by neurons, which was not the case with TLR3 deficient animals (De Miranda et al., 2010). And lastly, it has been shown that poly I:C treatment of pregnant rat dams leads to an impaired postnatal neurogenesis, but not disturbed microgliogenesis (Piontkewitz et al., 2012), as well as to an impaired adult neurogenesis (Zhang and van Praag, 2015), in the hippocampus of the offspring. Thus, poly I:C might well exert direct influence on neuronal development as proposed. However, this interaction can hardly explain the poly I:C induced cerebral immune-inflammatory response in the offspring. And this brings us back to the initial statement of Smolders and co-workers, namely, that microglia cannot be a main player in poly I:C induced developmental deficits. Assuming that this supposition is correct (some aforementioned *in vitro* and *in vivo* studies argue against this conjecture) one has to ask which brain tissue component then is to blame for the observed alterations, especially for the immune response? A “hot candidate” for this is astroglia. Astrocytes are abundantly populated with TLR3 (Farina et al., 2005, 2007; Park et al., 2006; Ibi et al., 2013; Ibi and Yamada, 2015 and others), become strongly activated after poly I:C and, most importantly in this context, are able to secret the whole battery of pro-inflammatory and anti-inflammatory cytokines, which are typically found after MIA with poly I:C (as reviewed by Ibi and Yamada, 2015). Moreover, when cultured neurons were incubated with the conditioned medium of poly I:C treated astrocytes, neurite development was found to be disturbed. This effect is mediated by an interferon-induced transmembrane protein 3, which is synthesized by, and released into the medium from, astrocytes after poly I:C treatment (Ibi et al., 2013). Analysis of conditioned media of astrocytes after poly I:C treatment subsequently revealed the presence of a further protein, matrix metalloproteinase 3, which also contributes to the observed impairment of neurite outgrowth and spine formation of cultured neurons. Of note, this protein is expressed in, and released from, astrocytes but not microglia (Yamada et al., 2014). Moreover, strong astroglial activation may be detected in postnatal hippocampi of the offspring after mid-gestational poly I:C MIA using GFAP immunolabeling (Ratnayake et al., 2012). Interestingly, Ibi and Yamada (2015) claim that poly I:C activates TLR3 in astrocytes of the brain parenchyma or BBB, thus pointing to a possible role of activated astroglia in impaired vascularization. Indeed, TRL3 activation has a pronounced anti-angiogenic effect (Grelier et al., 2013), but it is yet not fully clear, if astroglia is implicated in this process. In any case, impaired vascularization was found by reduced RECA-1 immunohistochemistry in postnatal rat hippocampi after MIA by poly I:C treatment (Piontkewitz et al., 2012). In sum, there are good reasons to consider astroglia as a major player in brain pathology of the offspring (including immune-inflammatory response) after maternal exposure to poly I:C.

## Author contributions

All authors listed, have made substantial, direct and intellectual contribution to the work, and approved it for publication.

### Conflict of interest statement

The authors declare that the research was conducted in the absence of any commercial or financial relationships that could be construed as a potential conflict of interest.
